# Cannabigerol Modulates Blood Pressure and Vascular Reactivity in a Rat Model of Hypertension and Platelet Responses in Human Blood In Vitro

**DOI:** 10.3390/molecules31193555

**Published:** 2026-10-06

**Authors:** Anna Krzyżewska, Krzysztof Mińczuk, Tomasz Misztal, Monika Kloza, Hanna Kozłowska

**Affiliations:** 1 Department of Experimental Physiology and Pathophysiology, Medical University of Białystok, Mickiewicz Str. 2A, 15-222 Białystok, Poland; krzysztof.minczuk@umb.edu.pl (K.M.); monika.kloza@umb.edu.pl (M.K.); hanna.kozlowska@umb.edu.pl (H.K.); 2 Department of Physical Chemistry, Medical University of Białystok, Mickiewicz Str. 2A, 15-222 Białystok, Poland; tomasz.misztal@umb.edu.pl

**Keywords:** cannabigerol, blood pressure, platelet function, vessels, vasorelaxant, hypertensive rats, pithed rats, hypotensive effect

## Abstract

Cannabigerol (CBG) exerts vasorelaxant effects in human pulmonary arteries and lowers blood pressure (BP) in normotensive mice. However, data on the cardiovascular effects of CBG in hypertension and platelet function remain limited. Consequently, the aims of our study were as follows: to assess the effect of CBG on BP and heart rate (HR) in spontaneously hypertensive rats (SHR) and their normotensive controls (Wistar Kyoto rats; WKY); to investigate the vasorelaxant potential of CBG in small mesenteric arteries (sMAs) and the aorta; and to investigate the effect of CBG on platelet function in human blood. Acute CBG administration (1, 3, and 10 mg/kg) dose-dependently lowered BP and HR in SHR and WKY rats, with comparable effects between strains (except for a greater reduction in systolic BP in SHR). Furthermore, experiments on pithed rats support the possibility of both central and peripheral contributions to this hypotensive effect. The peripheral component is supported by CBG-induced vasorelaxation in sMAs and aortas, which was more pronounced in sMAs and in WKY rats. Additionally, we demonstrated that CBG inhibits platelet aggregation (collagen-induced) and secretion and reduces thrombus formation and platelet procoagulant response under arterial flow conditions. Taken together, these data indicate that CBG exerts multifaceted cardiovascular effects.

## 1. Introduction

Cannabinoids are defined as a group of cannabinoid receptor (CB-R) ligands that can regulate a variety of bodily functions. They are classified into: endogenous compounds, such as anandamide (AEA) and 2-arachidonoylglycerol (2-AG), which are part of the endocannabinoid system (ECS); plant-derived compounds, for example, cannabidiol (CBD) and cannabigerol (CBG); and synthetic cannabinoids, like JWH-018. Given the extensive evidence demonstrating the widespread distribution of the ECS throughout the cardiovascular system, it is thought to play an important role in the regulation of cardiovascular function, particularly under pathological conditions [1].

Cannabigerol does not produce psychoactive effects, and there is a body of evidence suggesting that it exhibits antioxidant, anti-inflammatory, and neuroprotective effects, indicating its potential for therapeutic applications [2]. Cascio et al. [3] demonstrated that CBG is a potent agonist of the alpha 2 adrenoceptor (α_2_-AR) (half-maximal effective concentration; EC_50_ = 0.2 nM). Studies conducted on normotensive mice have shown that CBG at doses of 3.3 and 10 mg/kg reduced mean blood pressure (MBP), and this effect is attenuated following administration of an α_2_-AR antagonist [4]. This suggests that CBG may regulate vascular tone and blood pressure (BP) by reducing noradrenaline release from the sympathetic nerve fibres [4,5]. Furthermore, in addition to its effect on BP, CBG relaxed human pulmonary arteries (hPAs) in a manner dependent on the vascular endothelium, and the vasorelaxant response was attenuated in patients with hypertension and hypercholesterolaemia [6]. CBG also has a significant effect on platelet function. A study conducted by Formukong et al. [7] demonstrated that CBG inhibited platelet aggregation induced by adenosine diphosphate in both rabbits and humans (Ki = 2.7 × 10^−4^ M), whilst Polanska et al. [8] observed that 90-day administration of CBG at doses of 0.66 mg/kg/day and 1.33 mg/kg/day led to a reduction in platelet count in rats, which may limit thrombus formation and thus could be significant in the prevention of cardiovascular events associated with hypertension.

However, whilst the effects of other phytocannabinoids, such as Δ^9^-tetrahydrocannabinol and CBD, on the cardiovascular system are well documented [9,10,11], those of CBG remain poorly understood. Despite the few reports mentioned above, the available data are limited, particularly in the context of hypertension. It is worth noting that hypertension is associated with endothelial dysfunction, altered shear stress, and increased platelet activation, which is associated with an increased prothrombotic state (the so-called thrombotic paradox of hypertension) and increases the risk of thrombosis [12,13].

Taking the above into consideration, the aims of our study were: 1/ to assess the effect of acute intravenous (*i.v.*) administration of CBG (1, 3 and 10 mg/kg) on BP and heart rate (HR) in rats with primary hypertension (spontaneously hypertensive rats, SHR) and their normotensive controls (Wistar-Kyoto (WKY) rats); 2/ to investigate the involvement of the central nervous system in the hypotensive effect in pithed SHR and WKY rats; 3/ to assess the effect of CBG on vascular tone in the aortas and small mesenteric arteries (sMAs) in SHR and WKY rats; 4/ to evaluate the effect of CBG on human platelet function.

## 2. Results

### 2.1. Influence of Cannabigerol on Blood Pressure and Heart Rate

In SHRs anesthetized with sodium pentobarbital, baseline values of systolic blood pressure (SBP), diastolic blood pressure (DBP) and MBP were higher compared to WKY rats, but HR did not differ between the groups. In pithed and vagotomised SHRs, baseline DBP, MBP and HR were significantly higher than in WKY rats (Table 1).

In anesthetized and pithed WKY and SHRs, a single acute *i.v.* administration of the CBG vehicle did not change SBP, DBP or MBP compared to baseline. In anesthetized and pithed WKY rats, the CBG vehicle did not affect HR. In SHRs, the CBG vehicle slightly reduced HR from baseline in both anesthetized (by about 1%; *p* < 0.001) and pithed (by about 2%; *p* < 0.001) rats (*t*-test for paired data).

**Figure 1:**
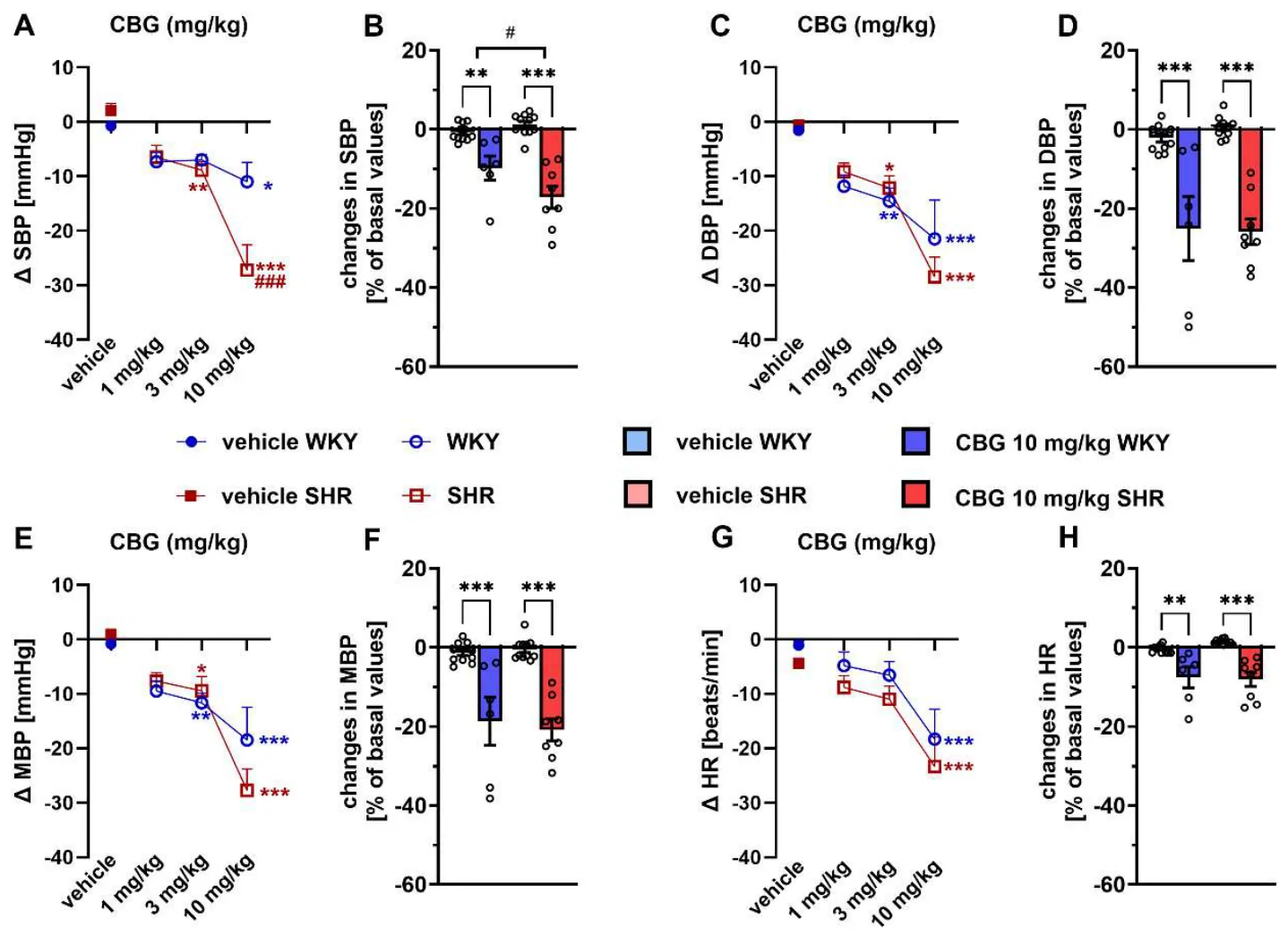
Comparison of the effects of cannabigerol (CBG) at three doses: 1, 3 and 10 mg/kg on systolic (SBP; (**A**)), diastolic (DBP; (**C**)), and mean (MBP; (**E**)) blood pressure, and heart rate (HR; (**G**)) in pentobarbital-anesthetized normotensive Wistar-Kyoto (WKY) rats and spontaneously hypertensive rats (SHR). The bar charts show the percentage changes in SBP (**B**), DBP (**D**), MBP (**F**) and HR (**H**) relative to baseline values for the highest dose of CBG (10 mg/kg). Data are presented as the mean ± SEM of 6–11 rats. # *p* < 0.05, ### *p* < 0.001 comparison between WKY and SHRs; * *p* < 0.05, ** *p* < 0.01, *** *p* < 0.001 comparison between vehicle vs. selected doses (one-way ANOVA followed by Bonferroni’s post hoc test). White circles represent individual data points.

In the anesthetized WKY and SHRs, CBG at doses of 1, 3 and 10 mg/kg caused dose-dependent reductions in SBP, DBP, MBP, and HR values, with the greatest effect observed at 10 mg/kg compared to the vehicle (Figure 1A,C,E,G). In WKY rats, the highest dose of CBG reduced SBP, DBP, MBP and HR values by about 11 mmHg (10%; *p* < 0.05), 22 mmHg (25%; *p* < 0.05), 19 mmHg (19%; *p* < 0.05) and 15 beats/min (5%; *p* < 0.05) compared to the baseline values, respectively. In SHRs, the same dose of CBG reduced SBP, DBP, MPB and HR by about 27 mmHg (18%; *p* < 0.001), 29 mmHg (26%; *p* < 0.001), 28 mmHg (21%; *p* < 0.001) and 23 beats/min (8%; *p* < 0.01) compared to the baseline values, respectively. It is worth noting that, when comparing the decreases in absolute values (mmHg or beats/min) and in percentages, only the reduction in SBP, following CBG (10 mg/kg) administration, was greater in SHR than in WKY rats (Figure 1A,B), while DBP, MBP and HR remained at comparable levels in both groups (Figure 1C–H).

**Figure 2:**
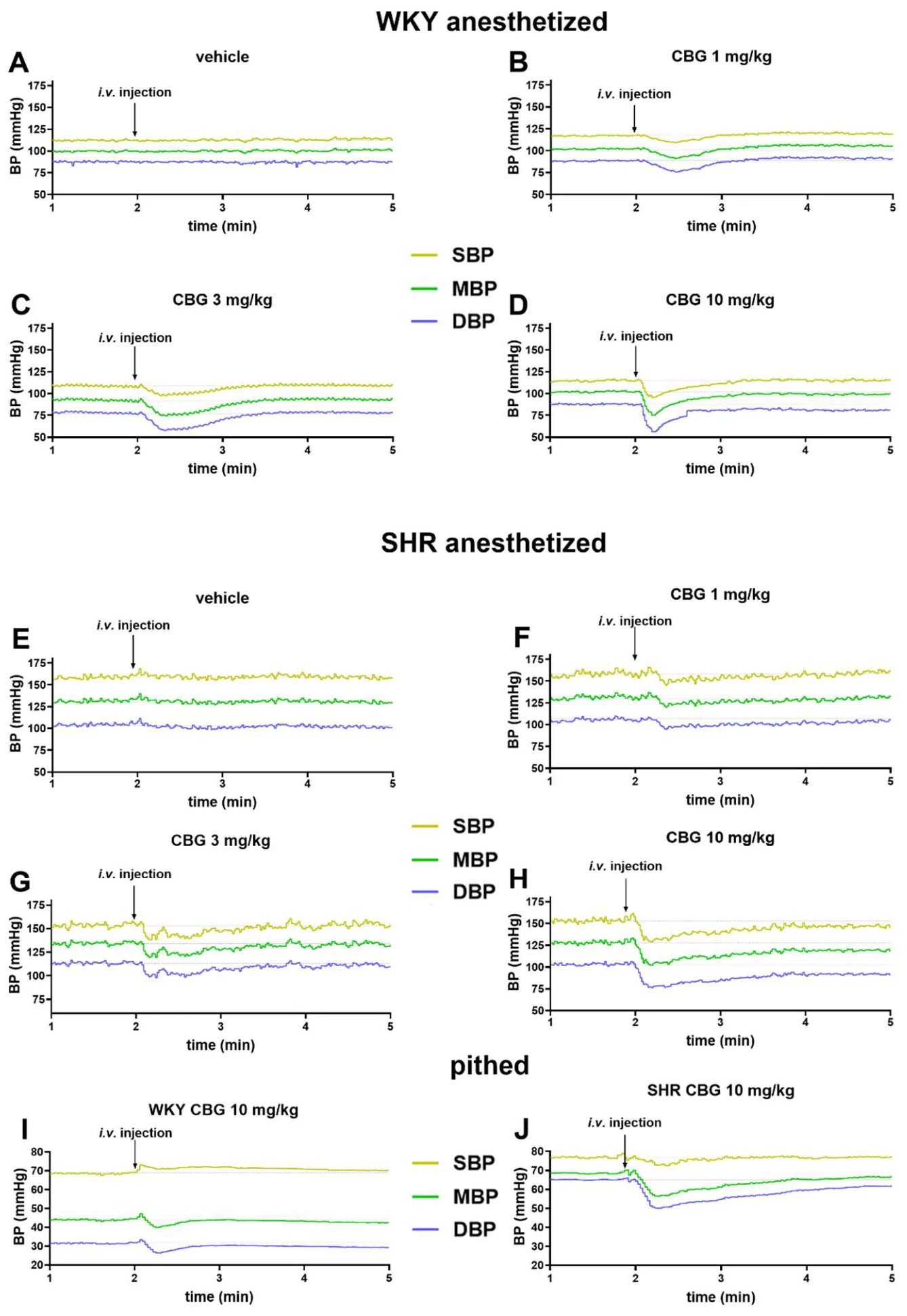
Traces from representative experiments showing the influence of vehicle (**A**,**E**) or cannabigerol (CBG) at doses of 1 (**B**,**F**), 3 (**C**,**G**), or 10 (**D**,**H**–**J**) mg/kg on systolic (SBP), diastolic (DBP) and mean (MBP) blood pressure in pentobarbital-anesthetized (**A**–**H**) and pithed and vagotomised (**I**,**J**) normotensive Wistar-Kyoto (WKY) rats and spontaneously hypertensive rats (SHR). Arrows show the moment of intravenous (*i.v.*) injection of the vehicle or CBG. Dotted lines indicate visual guides added to facilitate the reading of the values.

In the anesthetized WKY rats, the maximal hypotensive effect of all three doses of CBG was observed immediately after drug administration, with BP returning to baseline values after approximately 81 ± 7 s (1 mg/kg), 95 ± 4 s (3 mg/kg), and 118 ± 39 s (10 mg/kg) (Figure 2B–D). In the anesthetized SHRs, the maximal effect was observed immediately after administration of any of the three doses, the same as in the WKY group. Blood pressure recovery to baseline values occurred within 79 ± 11 s (1 mg/kg) and 106 ± 15 s (3 mg/kg) (Figure 2F,G). At a dose of 10 mg/kg, BP values did not return to baseline (observations were made for 10 min), and the MBP stabilized after approximately 112 ± 18 s and remained 8 mmHg (6%) lower than baseline at the end of the observation period (*p* < 0.05) (Figure 2H).

**Figure 3:**
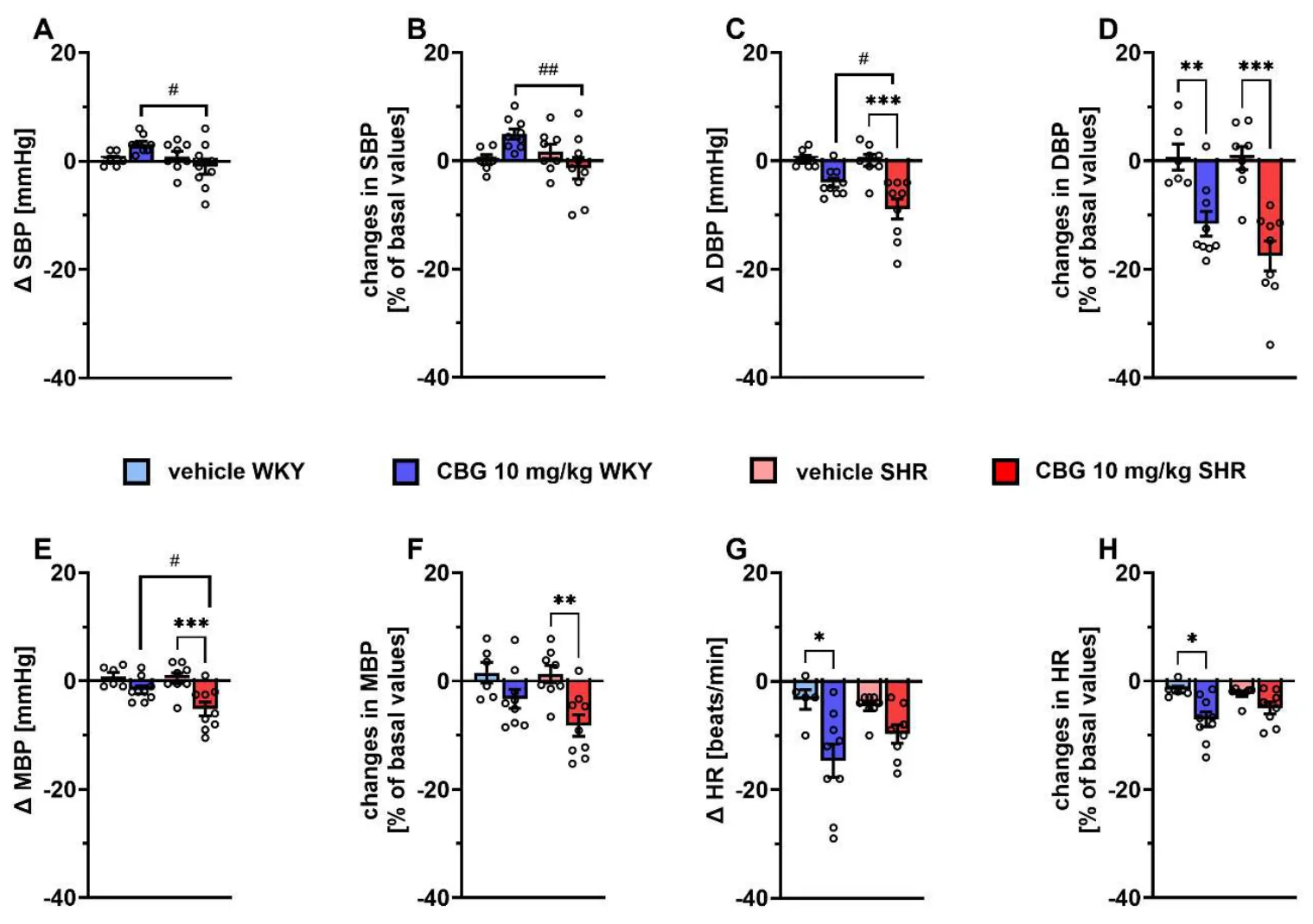
Effect of cannabigerol (CBG; 10 mg/kg) on systolic (SBP; (**A**,**B**)), diastolic (DBP; (**C**,**D**)) and mean (MBP; (**E**,**F**)) blood pressure, and heart rate (HR; (**G**,**H**)) in pithed and vagotomised normotensive Wistar-Kyoto (WKY) rats and spontaneously hypertensive rats (SHR). The bar charts (**A**,**C**,**E**,**G**) show changes in absolute values (mmHg or beats/min), and (**B**,**D**,**F**,**H**) show the percentage changes in blood pressure and HR relative to baseline values. Data are presented as the mean ± SEM of 6–11 rats. * *p* < 0.05, ** *p* < 0.01, *** *p* < 0.001 compared to the vehicle; # *p* < 0.05, ## *p* < 0.01 comparison between WKY and SHRs. For comparison, one-way ANOVA followed by Bonferroni’s post hoc test was used. White circles represent individual data points.

In the pithed rats, the effect of CBG (10 mg/kg) on BP was different from that in anesthetized rats. In this group, there was a tendency for SBP to rise (especially in WKY rats), while DBP decreased in both WKY and SHRs compared with baseline values by about 4 mmHg (12%; *p* < 0.01) and 9 mmHg (19%; *p* < 0.01), respectively. CBG significantly reduced HR only in WKY rats by about 11 beats/min (4%; *p* < 0.001). In pithed and vagotomised WKY rats CBG at a dose of 10 mg/kg, when comparing the changes in absolute values (mmHg or beats/min), only HR values decreased compared to the vehicle (Figure 3G). However, when converting the values to percentage, CBG decreased DBP and HR compared to the vehicle (Figure 3D,H). In the SHR group, the same dose of CBG decreased (both changes in absolute values and in percentage values) DBP and MBP compared to the vehicle (Figure 3C–F). When comparing the changes after CBG administration in absolute values (mmHg or beats/min), the SHR group recorded greater decreases in SBP, DBP, and MBP than WKY rats. When comparing the percentage changes in values, no differences were observed between WKY and SHRs, except for SBP, where, following administration of CBG, WKY rats exhibited higher SBP values than SHRs (Figure 3B,D,F,H). The maximal hypotensive effect of CBG was observed immediately after drug administration in both WKY and SHRs (see Figure 2I,J).

### 2.2. Influence of Cannabigerol on Vascular Response

The vasorelaxant effects of CBG were evaluated in sMAs and aortas precontracted with phenylephrine (10 μM and 0.3 μM, respectively), corresponding approximately to its EC_60_. The magnitude of phenylephrine-induced contraction was comparable between SHR and WKY vessels. In sMAs, contractions reached 11.8 ± 2.5 mN (*n* = 12) in SHRs and 11.2 ± 2.2 mN (*n* = 11) in the WKY group. In aortas, the respective values were 7.4 ± 0.7 mN (*n* = 12) and 7.5 ± 0.4 mN (*n* = 8), while in the ethanol control group, the contraction was 7.0 ± 0.7 mN (*n* = 7). The α_1_-AR agonist (−)-phenylephrine (0.001–30 µM) induced a concentration-dependent contraction of aortic rings of WKY rats (pEC_50_ = 6.8 ± 0.1, *n* = 8). Cannabigerol (10 μM) did not modify the cumulative concentration–response curve (CRC) of phenylephrine (pEC_50_ = 6.7 ± 0.1, *n* = 8).

**Figure 4:**
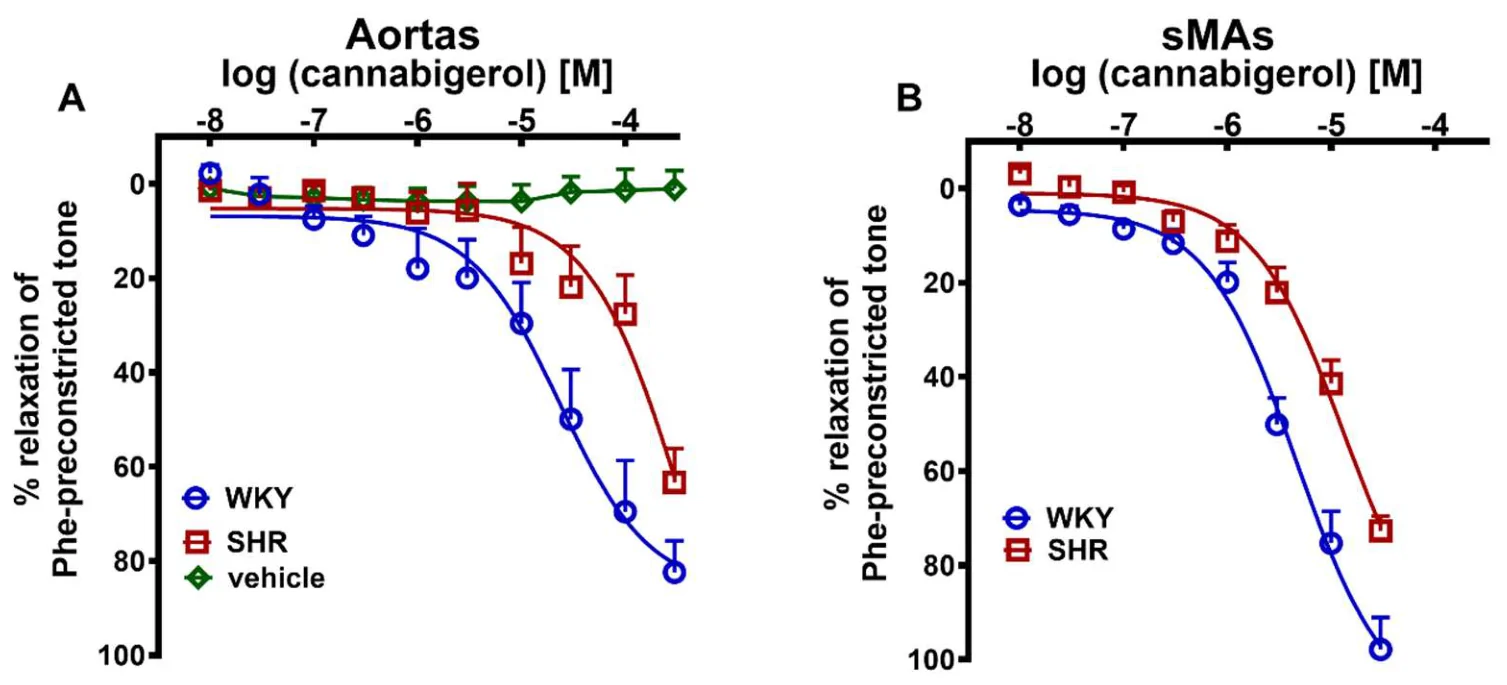
Cumulative concentration–response curves for cannabigerol (CBG)-induced relaxation in the aortas (**A**) and small mesenteric arteries (sMAs; (**B**)) from normotensive WKY and hypertensive SHRs. Ethanol (vehicle) produced no relaxation. Responses are expressed as a percentage of phenylephrine (Phe)-induced contraction in endothelium-intact vessels. Data are presented as mean ± SEM (*n* = 8–12 rings).

CBG, but not its vehicle (ethanol; 0.0005–0.7%), produced concentration-dependent relaxation in isolated arteries from both strains (Figure 4A). In aortas, maximal response (R_max_) reached 63.3 ± 5.1% (*n* = 12) in SHR and 82.4 ± 6.5% (*n* = 8) in WKY (*p* < 0.05), with corresponding pEC_50_ values of 3.5 ± 0.2 and 4.6 ± 0.2 (*p* < 0.01) (Figure 4A).

In sMAs, CBG induced stronger relaxation than in aortas. The R_max_ values were 72.6 ± 3.1% (*n* = 12) in SHR and 97.9 ± 6.8% (*n* = 11) in WKY (*p* < 0.05), with pEC_50_ values of 4.9 ± 0.2 and 5.4 ± 0.1 (*p* < 0.01) (Figure 4B). The vasorelaxant effect of CBG was stronger in normotensive WKY rats than in the SHR group, both in the aorta and in the sMAs (see pEC_50_).

### 2.3. Influence of Cannabigerol, Cannabidiol and Their Combination on Platelet Function

**Figure 5:**
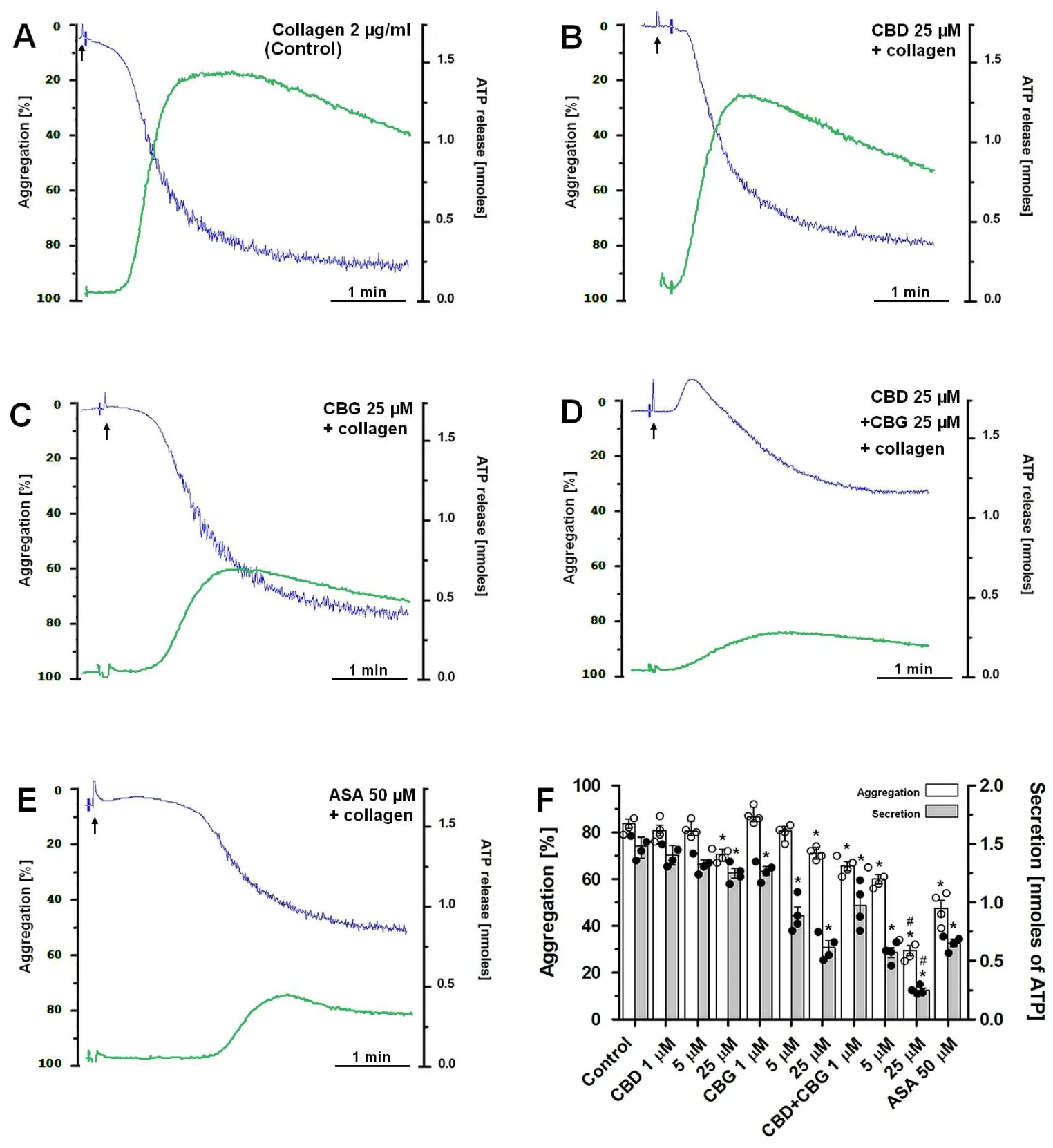
Effect of cannabidiol (CBD), cannabigerol (CBG) and their combination on collagen-induced platelet aggregation and adenosine triphosphate (ATP) secretion. PRP samples were incubated for 5 min with indicated concentrations of CBD, CBG, their combination, or vehicle (ethanol in control samples) in an aggregometer cuvette, in the presence of luciferin-luciferase reagents. Afterwards, collagen was added (arrow) to trigger platelet aggregation and secretion. Optical aggregation (blue traces) was measured simultaneously with ATP secretion (monitored luminometrically, green traces) from activated platelets. Representative traces in control (**A**) and in samples preincubated with indicated concentrations of CBD (**B**), CBG (**C**), their combination (**D**), and a positive antiplatelet control, acetylsalicylic acid (ASA, cyclooxygenase-1 inhibitor, 50 μM, (**E**)) are presented. Median values (with a range of 1st–3rd quartile) from 4 independent experiments (each performed on platelets collected from different donors, in three technical repetitions) of aggregation degree (in %) and ATP release (in nanomoles) are presented in graph (**F**). Each test was performed in at least two technical repetitions. *n* = 4; * *p* < 0.05 vs. control; # *p* < 0.05 vs. ASA. Black circles represent individual values for secretion, whereas white circles represent individual values for aggregation. Blue lines indicate aggregation, and green lines indicate ATP release.

CBD and CBG (alone) dose-dependently (1–25 µM) inhibited the aggregation of platelets in platelet-rich plasma (PRP) evoked by collagen (*p* < 0.05 at 25 µM for both cannabinoids, Figure 5A–C). When added in combination, the enhancement of the antiaggregatory effect was observed within the 1–25 µM concentration range of these cannabinoids (*p* < 0.05 at 1–25 µM, Figure 5D). Along with the inhibitory effect of CBD, CBG and their combination on platelet aggregation, secretion from platelet dense granules (measured as adenosine triphosphate (ATP) release) was also significantly reduced, with the more distinct effect observed for CBG (*p* < 0.05 at 1–25 µM) than for CBD (*p* < 0.05 at 25 µM). When combined, CBD and CBG strongly inhibited platelet aggregation and ATP secretion triggered by collagen (*p* < 0.05 at 1–25 µM). The combination of 25 µM concentrations for each CBD and CBG exerted stronger anti-aggregatory and anti-secretory effects than ASA (50 µM, Figure 5E,F).

**Figure 6:**
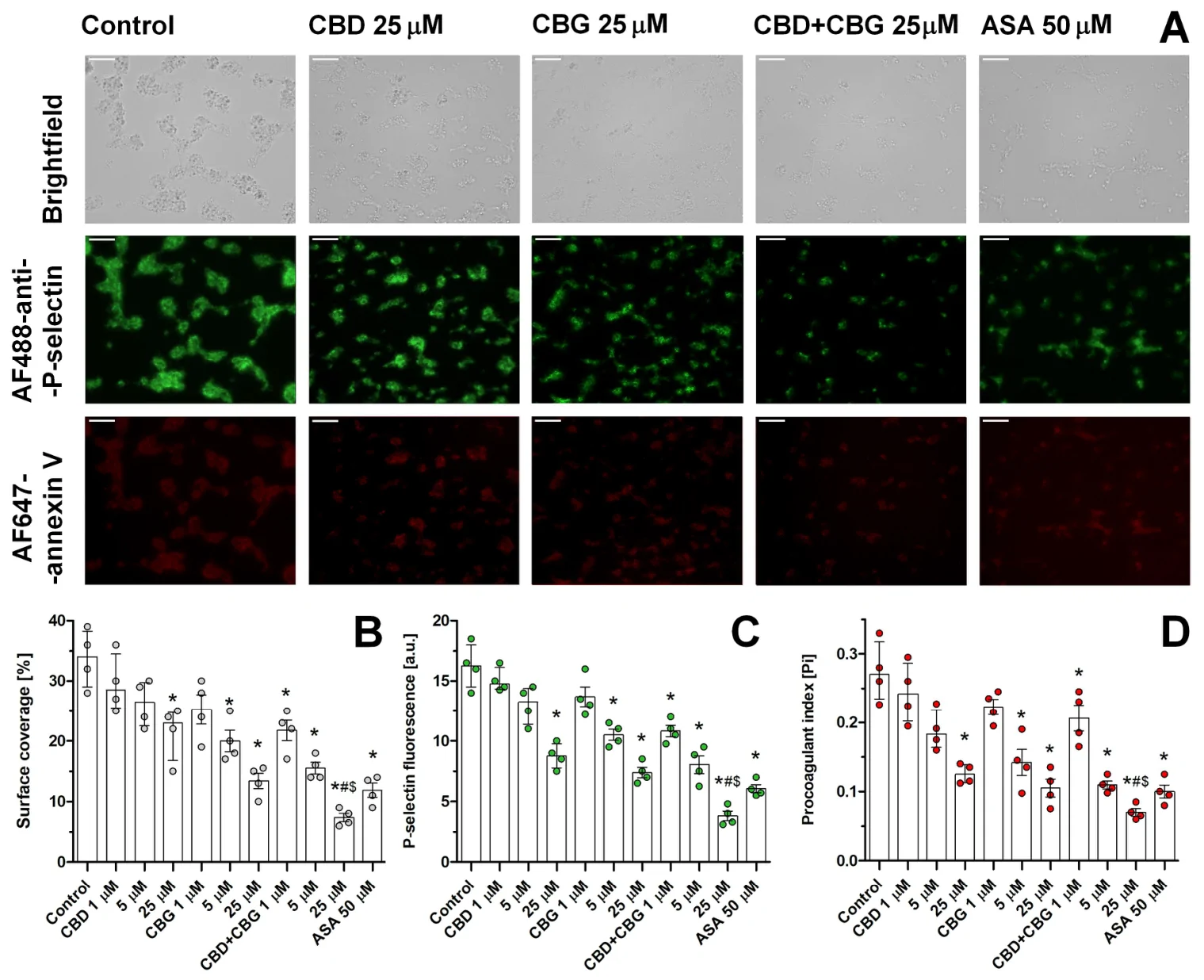
Effect of cannabinoids on platelet thrombi formation, P-selectin secretion and procoagulant response under arterial flow. PPACK-anticoagulated whole blood samples were preincubated for 5 min with indicated concentrations of CBD, CBG, their combination, or vehicle (ethanol in control samples), followed by perfusion through the collagen-coated parallel-plate flow chamber at arterial shear rate (1000 s^−1^). Resulting platelet thrombi were stained for P-selectin exposure (AF488, green fluorescence) and annexin V binding (AF647, red fluorescence) and also collected in brightfield mode (**A**). The effects of studied cannabinoids were compared with a positive control, acetylsalicylic acid (ASA, cyclooxygenase-1 inhibitor, 50 μM). Effect of studied compounds on platelet adhesion (**B**), secretion (**C**), and procoagulant index (**D**) are presented as median values with interquartile range (1st–3rd). *n* = 4, * *p* < 0.05 vs. control; # *p* < 0.05 vs. CBG 25 μM; $ *p* < 0.05 vs. ASA. Distance bar = 20 μm. White circles represent individual surface coverage values, green circles represent individual P-selectin fluorescence values, and red circles represent individual procoagulant index values.

To determine whether CBD and CBG may modulate platelet adhesion to a collagen-coated surface, subsequent activation, and thrombus formation under conditions mimicking arterial flow, we utilized a flow chamber device coupled with a fluorescent microscope to acquire platelet activation status. Incubating whole blood samples with CBD (25 µM) or CBG (5–25 µM) resulted in a significant (*p* < 0.05) reduction in platelet adhesion to collagen-coated surfaces under an arterial (1000 s^−1^) shear rate. Notably, when combined, CBD + CBG exerted a dose-dependent inhibitory effect (*p*< 0.05 at 1–25 µM), which—at 25 µM concentration of both compounds—was stronger than the effect of ASA (Figure 6A,B). A similar pattern of CBD/CBG-evoked inhibition was observed in the case of platelet secretion (measured as P-selectin exposure on the surface of collagen-deposited platelets, Figure 6A,C) and procoagulant response (measured as binding of annexin V to phosphatidylserine, exposed on the outer bilayer of the plasma membrane of procoagulant platelets, Figure 6A,D).

## 3. Discussion

### 3.1. General

The aims of this study were to investigate whether a single acute *i.v.* administration of CBG (at doses of 1, 3 and 10 mg/kg) affects cardiovascular parameters in rats with primary hypertension (SHR) and their normotensive controls (WKY), as well as to assess the involvement of the central nervous system in the effect of CBG in pithed and bilaterally vagotomised rats (in which, due to spinal cord injury, only peripheral mechanisms can be studied) [14]. Additionally, we assessed the effect of CBG on vascular tone of the aorta and sMAs (transport vs. resistance vessels) in SHR and WKY rats and initially evaluated the effect of CBG on human platelet function in in vitro studies.

As approximately 90–95% of cases of hypertension in humans are classified as primary hypertension, we used the male SHR strain in our study. This is a polygenic model, one of the most widely used in research, in which most antihypertensive drugs are effective, and the pathophysiological changes are similar to those observed in primary hypertension in humans [15,16]. We conducted experiments on rats anesthetized with sodium pentobarbital because it does not impair the function of presynaptic receptors, which, according to our findings following a review of the literature, may mediate the hypotensive effect of the compound we are investigating [17].

### 3.2. Cannabigerol Lowers BP and HR in Normotensive and Hypertensive Conditions

In our study, we demonstrate for the first time that a single *i.v.* administration of CBG reduces SBP, DBP, MBP and HR in SHRs and their normotensive WKY controls, and that these effects are dose-dependent, being greatest at a dose of 10 mg/kg. Previous data on the effects on BP (still limited to a single study) involved only normotensive animals, with no data from the SHR model [4]. In our study, we compare the WKY and SHR strains, which allows us to assess whether the hemodynamic effect persists, weakens, or changes in nature in the context of already elevated blood pressure. This is clinically significant given the prevalence of hypertension in the general population.

As mentioned, to date, a single study has been conducted to assess the direct effect of CBG on cardiovascular parameters, in which Vernail et al. [4] demonstrated that a single intraperitoneal (*i.p.*) administration of CBG (at doses of 3.3 and 10 mg/kg) reduced MBP (with the maximum effect occurring after approximately 90 min) in normotensive mice. This effect was not dose-dependent and was abolished by the administration of an α_2_-AR antagonist [4], suggesting that the hypotensive effect of CBG may be associated with the activation of presynaptic α_2_-AR autoreceptors, leading to the inhibition of noradrenaline release from the terminals of sympathetic neurons innervating the heart and blood vessels [18]. Unlike previous studies [4], which analyzed the effects of CBG following *i.p.* administration, in the present study we used *i.v.* administration, which eliminates the influence of absorption processes from the peritoneal cavity and allows for a more precise assessment of the relationship between dose and cardiovascular response.

Our findings regarding the hypotensive effect of CBG are consistent with the study conducted by Vernail et al. [4]. However, in the study in question, the authors did not observe the expected compensatory increase in HR in response to a decrease in BP in mice [4]. Importantly, rats have a lower resting HR than mice, so it cannot be ruled out that CBG may modify HR in species with a lower resting HR [10]. Furthermore, in our study, following *i.v.* administration of CBG at the highest dose, we observed a slight decrease in HR (in both SHR and WKY rats) alongside a decrease in SBP and DBP. Therefore, we conducted part of the study on pithed and bilateral vagotomised rats to assess the direct effects of CBG, independently of central nervous system and reflex responses. Our studies demonstrate for the first time that, even after disrupting central regulation, a significant portion of the hemodynamic effect may arise from peripheral mechanisms rather than solely from effects on the central nervous system. We demonstrated that the observed decrease in DBP following CBG administration (10 mg/kg) in pithed and vagotomised rats indicates a direct vasorelaxant effect of CBG, leading to a reduction in vascular resistance. In anesthetized rats after CBG administration, we observed a decrease in DBP of 25% in WKY rats and 26% in SHRs, and in pithed and vagotomised rats we observed a decrease in DBP of 12% in WKY and 19% in SHRs, respectively. The smaller decrease in DBP observed in pithed rats compared with anesthetized rats may reflect their lower baseline DBP, representing a limitation of the study. Therefore, in order to confirm the peripheral component, we carried out additional studies on isolated vessels, and the vascular effects are described in the section below.

The slight decrease in HR in WKY pithed and vagotomised rats following CBG administration may suggest a direct effect of CBG on the heart. However, given the discrepancies in the effects on HR between the study by Vernail et al. [4] and our own study, further research should be conducted to examine the direct effect of CBG on the heart with reference to studies involving rats with and without hypertension. The lack of decrease in SBP following CBG administration in pithed rats may indicate that the earlier decrease (in anesthetized rats) was partly dependent on central mechanisms.

In our study, we observed that the SBP-lowering effect of CBG in anesthetized SHRs was more pronounced than in WKY rats. We hypothesize that this may result from (a) higher baseline BP in SHRs and (b) increased sympathetic activity and impaired parasympathetic function in SHRs [19], which may exacerbate the response to CBG in SHRs.

### 3.3. Cannabigerol Relaxes Aortas and sMAs in Normotensive and Hypertensive Conditions

As one of our hypotheses was that CBG may lower DBP partly through direct vasorelaxant effects and by reducing blood flow resistance, we conducted part of our studies on isolated vessels. For these studies, we used aortas, which primarily serve a conductive function, and sMAs, which are resistance vessels and regulate BP [20]. In this study, we demonstrate for the first time the direct vasorelaxant effect of CBG on the sMAs and aortas of SHRs and their normotensive WKY controls, providing new evidence for its vascular effects under hypertensive conditions. This finding is clinically relevant because vascular tone regulation is often altered in hypertension, a condition that is highly prevalent in the general population. Based on the analysis of pEC_50_ and R_max_ values, CBG elicited a greater vasorelaxant response in sMAs than in aortas, which suggests that CBG preferentially targets resistance vessels and this effect is more pronounced in normotensive rats. Since isolated vessels lack functional sympathetic innervation, the vasorelaxant response observed in our ex vivo experiments cannot be explained by presynaptic α_2_-ARs activation. Therefore, our findings indicate the presence of a direct peripheral vascular mechanism, independent of presynaptic α_2_-ARs. Importantly, the lack of effect of CBG on the phenylephrine-induced contractile response suggests that CBG does not functionally interact with α_1_-ARs.

We hypothesize that the weaker vasorelaxant response of sMAs to CBG in SHRs than in WKY rats may be due to the well-documented endothelial dysfunction and/or structural changes in the vascular wall characteristic of hypertension [20,21]. It should be noted, however, that the studies were conducted exclusively on vessels with intact endothelium (i.e., the selection of vessels with ≥90% acetylcholine-induced relaxation), which makes it impossible to clearly distinguish between the contribution of the endothelial component and the direct effect on smooth muscle. This is further supported by the fact that in our previous studies we demonstrated that the potency of CBG was significantly reduced in hPAs derived from patients with hypertension [6]. Taking the pEC_50_ values into account (approximately 4.9 for SHR and 5.4 for WKY), CBG exhibited a weaker vasorelaxant effect on sMAs compared with other cannabinoids, including, for example, CBD, for which the pEC_50_ value was 5.1 in human mesenteric arteries [22], 6.0 in sMAs from WKY rats and 5.6 in sMAs from SHRs [23].

### 3.4. Cannabigerol Inhibits Platelet Aggregation and Secretion, and Reduces Thrombus Formation and Platelet Procoagulant Response Under Arterial Flow Conditions

To initially assess the effects of CBG on the platelet haemostatic response in human blood, we investigated whether CBG modifies platelet aggregation, secretion (providing secondary platelet agonists), and thrombus formation under conditions mimicking arterial flow, all triggered by the principal physiological platelet agonist, type I collagen. In addition, CBD was included as a comparator compound due to its structural similarity to CBG (can structurally be considered the cyclohexenyl-opened analogue of CBD) [24] and previously reported effects on platelet function [7]. Our study demonstrates, for the first time, that CBG, alone and in combination with CBD, reduced early platelet responses (secretion and aggregation), as well as thrombus formation under flow. This was expressed by the reduced adhesion of platelets to collagen, their subsequent activation and secretion, and by diminished platelet deposition and exposure of procoagulant phosphatidylserine (PS). Of importance, our preliminary examinations showed that the combination of 25 μM CBD and 25 μM CBG produced an inhibitory effect, which was of a similar magnitude to that exerted in vitro by a classical antiplatelet agent, ASA (50 μM). The key point is that this part of the experiments was conducted using patients’ blood, rather than animal blood. This approach has greater translational value because it shows that the antiplatelet effect is not limited to the rodent model.

While a study by De Angelis et al. [25] evidenced that platelets obtained from Cannabis sativa consumers showed reduced ability to aggregate (triggered by collagen), the exact molecular mechanism remained unknown. The preliminary results presented here indicate that CBG may be responsible, at least partially, for the antiplatelet effects of Cannabis sativa and advocate for further searching for molecular targets for CBG in platelets. CBG has been shown to inhibit a purified cyclooxygenase-1 (abundantly expressed in platelets), the key enzyme engaged in the thromboxane A_2_ (a potent secondary platelet agonist) synthesis pathway. This is in line with the report of Formukong et al. [7], where CBG has been shown to inhibit platelet aggregation of human and rabbit platelets induced by ADP and adrenaline, i.e., agonists whose mechanism of action strongly depends on thromboxane A_2_ formation. Taking all these reports into account, CBG has an antithrombotic effect under conditions of arterial blood flow, suggesting its potential significance in the prevention or treatment of thromboembolic diseases; however, the complex effect of CBG on platelet signalling still awaits unravelling.

## 4. Materials and Methods

### 4.1. Animals

All experimental procedures were approved by the Local Ethical Committee for Animal Experiments in Olsztyn (No. 09/2024, date of issue: 24 January 2024) and were conducted in accordance with Directive 2010/63/EU and the ARRIVE guidelines 2.0, considering the 3Rs principle (replacement, refinement, reduction). The animals were obtained from the Central Laboratory of Experimental Animals (Preclinical Research Centre; Medical University of Warsaw, Poland). The study was performed on 60 male WKY (control) rats and 60 male SHRs (aged 10–12 weeks, body weight 290–320 g). The animals were kept in plastic cages (two individuals per cage) under controlled environmental conditions: 12 h light/12 h dark cycle, temperature of 22 ± 2 °C and humidity of 50%, with unlimited access to tap water and standard granulated feed.

### 4.2. Surgical Preparation of Anesthetized and Pithed Rats

After a three-day acclimatization period, the animals were randomly assigned to the appropriate experimental groups. Before the main experiments, SBP was measured in conscious WKY and SHRs using a non-invasive tail-cuff method with the Non-Invasive Blood Pressure Controller (Hugo Sachs Elektronik-Harvard Apparatus, March-Hugstetten, Germany). Only SHRs with an SBP exceeding 150 mmHg were classified as hypertensive. The rats were anesthetized *i.p.* with pentobarbitone sodium (300 µmol/kg or ~70 mg/kg). A subset of rats was randomly allocated to the spinal cord injury (pithed rats) group. To this end, after anesthesia (pentobarbitone sodium) and tracheal incision, the animals were ventilated (parameters: 10 mL/kg; 60 strokes/min) using a ventilator (7025 rodent ventilator, Ugo Basile, Comerio, Italy). A stainless-steel rod was inserted through the orbit and great aperture into the spinal canal to damage the medulla oblongata and spinal cord (hence destroying the central nervous system). In addition, rats were bilaterally vagotomised [14,26]. No significant bleeding requiring intervention was observed during the procedure. Following pithing, mechanical ventilation was continued throughout the experiment to maintain respiration.

To measure cardiovascular parameters in both anesthetized and pithed rats, including BP and HR, the left carotid artery was exposed and separated from the surrounding tissues. Next, polyurethane catheters filled with heparinised saline solution (150 IU/mL) were inserted into the carotid artery to prevent blood clot formation within the catheters. Additionally, a cannula was inserted into the right femoral vein to enable *i.v.* administration of the substances (CBG or vehicle) at a volume of 0.5 mL/kg [26].

### 4.3. Experimental Protocol

After completion of the surgical procedures, the animals were left for a period of 15–30 min until stable haemodynamic parameters were achieved, after which the actual experimental protocol was carried out. During each phase of the experiment, cardiovascular parameters were monitored via the left carotid artery using a pressure transducer system (model MLT844, ADInstruments, Sydney, Australia) with computer data acquisition (Bridge Amp/PowerLab 4/35, ADInstruments) [27]. Only rats that exhibited stable baseline cardiovascular parameters were included in the experiment. Animals in which stable values of the BP parameters under investigation were not obtained during the experiment were excluded from the analysis, which affected the final size of the individual groups. However, the minimum group size was set at 6 animals. The sample size was based on our experience in this field and the expected variability of the measured parameters. Cannabigerol (1, 3 and 10 mg/kg) or its vehicle (1:1:18 DMSO: Tween80: saline) was injected *i.v.* and BP and HR parameters were monitored. Each dose of CBG or its vehicle was administered at a volume of 0.5 mL/kg and tested on a separate group of rats. This dose range was chosen based on other studies that have shown CBG’s positive effects. In addition, a single administration of CBG at the doses of 3.3 and 10 mg/kg was effective in lowering BP in normotensive mice [4]. In pithed and vagotomised rats, only a single dose of CBG (10 mg/kg) was tested. The allocation of animals to experimental protocols, groups and cage locations, as well as the order in which procedures and measurements were carried out, was randomized. The researchers were aware of the allocation of animals both during the experiments and during the statistical analysis.

### 4.4. Vessel Preparation

Male SHR and WKY rats were anesthetized with pentobarbitone sodium (300 µmol/kg or ~70 mg/kg). The thoracic aorta and third-order branches of the main mesenteric artery were isolated and cleaned of surrounding connective tissue. Rings from the thoracic aorta (3–5 mm in length) and 2 mm segments from sMAs were prepared for isometric tension measurements. sMA segments were mounted in a Mulvany-Halpern wire myograph (MultiWire Myograph System DMT 620 M, Danish Myo Technology, Aarhus, Denmark), whereas aortic rings were placed in 10 mL organ baths (PIM 100RE, BIO-SYS-TECH, Białystok, Poland), as previously described [28]. All vessels were maintained at 37 °C in Krebs–Henseleit solution (in mM: NaCl 118, KCl 4.8, CaCl_2_ 2.5, MgSO_4_ 1.2, NaHCO_3_ 24, KH_2_PO_4_ 1.2, glucose 11, and EDTA 0.03; pH 7.4), continuously gassed with 95% O_2_ and 5% CO_2_. Vessels were allowed to equilibrate for 60 min (resting tension ~14.7 mN) for thoracic aortas and for 30 min (resting tension ~5.5 mN) for sMAs. Endothelial function was verified by precontraction with the α_1_-adrenoceptor agonist (−)-phenylephrine (0.3 μM in aortas and 10 μM in sMAs) followed by acetylcholine-induced relaxation (Ach; 1 μM and 10 μM, respectively). Rings showing ≥90% relaxation were considered endothelium intact. In these experiments, Ach served as a positive control for endothelial-dependent relaxation and tissue viability. A specimen was considered suitable for further investigation only if Ach-induced marked relaxation of the pre-contracted ring, confirming preserved endothelial integrity and function. Rings that did not meet these criteria were excluded. After washout and stabilization, all vessels were constricted sub-maximally with phenylephrine and when a stable level of tone was maintained, CRCs were obtained by CBG or vehicle (ethanol). To exclude any effect of the solvent, vehicle-control experiments were performed in parallel, confirming that the vehicle itself did not induce vasorelaxation. To examine the potential effect of CBG on vasoconstriction increasing concentrations of phenylephrine in aortas some rings were treated for 30 min with one concentration (10 µM) of CBG and then a CRC for phenylephrine were constructed.

### 4.5. Blood Collection and Preparation

Venous blood was collected from healthy volunteers (two females, two males, age: 32–42) with minimal trauma and stasis via a 21-gauge needle (0.8 × 40 mm) into 10 mL polypropylene tubes containing 1 mL of 130 mM (3.2%) trisodium citrate (S-monovette, Sarstedt, Nümbrecht, Germany). All procedures were conducted in accordance with the principles of the World Medical Association Declaration of Helsinki, and the study was approved by the local Ethics Committee on human research (APK.002.181.2024; date of consent: 21 March 2024). The privacy rights of human subjects have been observed, and written informed consent was obtained from all the blood donors. The exclusion criteria comprise anticoagulant or anti-platelet therapy for at least 4 weeks, intake of non-steroidal anti-inflammatory drugs for 2 weeks before blood collection, alcohol consumption for 72 h before blood collection, and diagnosed congenital disorders associated with platelet-dependent or plasma-related hemostasis. PRP was obtained by centrifugation of whole blood at 200× *g* for 20 min. To prepare platelet-poor plasma (PPP), PRP was centrifuged at 2800× *g* for 10 min.

### 4.6. Assessment of Platelet Aggregation and ATP Secretion

Platelet aggregation in PRP (triggered by collagen) was measured optically as an increase in light transmittance, associated with aggregation progress was measured using a Chrono-log^®^ 700 lumiaggregometer (Chrono-log Corp., Haverton, PA, USA). At the same time, luminometric signals corresponding to ATP release from platelet dense granules were quantified. A high-gain photomultiplier recorded the light emitted during the reaction between platelet-released ATP and the firefly luciferin-luciferase mixture (CHRONO-LUME^®^ reagent), which was proportional to the amount of secreted ATP (expressed in nanomoles) following the addition of the stimulator (collagen, 2 μg/mL final conc., CHRONO-PAR^®^ reagent), according to the manufacturer’s protocol. Measurements were conducted at 37 °C up to 5 min to obtain maximal response.

### 4.7. Evaluation of Platelet Thrombus Formation, Secretion, and the Procoagulant Index Under Arterial Flow Conditions

Thrombus formation under flow was assessed as described by de Witt et al. [29]. Degreased (using 2 M HCl in 50% ethanol) coverslips, coated with collagen (type I, CHRONO-PAR^®^ reagent, 50 μg/mL), and microspots (1 mm in diameter) were mounted onto a transparent, polycarbonate-made parallel-plate flow chamber (50 μm in depth, 3 mm in width and 30 mm in length) and pre-rinsed with Hepes buffer (pH 7.45) with the addition of 0.1% bovine serum albumin (BSA). Freshly collected trisodium citrate-anticoagulated whole blood samples were supplemented with PPACK (40 μM to inhibit thrombin activity). Next, the samples (500 μL) were supplemented with MgCl_2_ and CaCl_2_ (the final concentrations of added MgCl_2_/CaCl_2_ in samples were 3 mM). Such prepared samples were incubated with vehicle (ethanol), CBD, CBG or their combination for 5 min at ambient temperature. Perfusions (using KDS Legato™-101 syringe pump, kdScientific, Holliston, MA, USA) were done through the flow chamber for a time sufficient for full thrombus formation on collagen microspots (4 min at arterial shear rate, i.e., 1000 s^−1^). Thrombi formed on collagen spots during the blood flow were post-stained by a 2 min perfusion (1000 s^−1^) with AlexaFluor (AF)488-anti-P-selectin antibody (final antibody dilution 1:20) and AF647-annexinV (5 μg/mL) in Hepes buffer pH (7.45), supplemented with 0.1% BSA and 2 mM CaCl2. After staining, the unbound label was removed by a short perfusion with the same Hepes buffer without labelled proteins. No fixative was used. The fluorescence images were generated using a fluorescence microscope (Celena S, Logos Biosystems, Anyang, Republic of Korea), equipped with a 40×/0.6 objective (TC PlanFluor, WD 2.9, Olympus, Tokyo, Japan), from randomly selected areas within collagen microspots. Focus was set manually by the operator. The surface coverage by adhered platelets was calculated with the use of ImageJ free software Version 1.54g [30]. To evaluate alpha granule secretion in platelets within thrombi, green fluorescence (associated with P-selectin) was acquired. The procoagulant index was calculated as the surface area of PS (annexin V)-positive platelets (red fluorescence) divided by the surface area of all adhered platelets from the considered location. At least 8 pictures from each microspot were collected. Each flow experiment was performed in technical duplicate.

### 4.8. Chemicals

The CBG was kindly provided by CB21 Pharma s.r.o. (successor to CBDepot). The composition and purity of the CBG have been determined and meet the specifications according to the European Pharmacopeia: Assay of Cannabigerol 98.0–102.0%. The analysis was performed following SOP 16.05 by E&H Services, confirming a purity of 99.7%. Cannabidiol (CBD; THC-1073G-1) was received from THC Pharm, Frankfurt, Germany. Tween 80 (P1754) was obtained from Sigma-Aldrich, Munich, Germany; pentobarbital sodium (5909991290153) from Biowet, Puławy, Poland and dimethyl sulfoxide (DMSO; D5879) from Sigma-Aldrich, Darmstadt, Germany. Stock solutions of phenylephrine and Ach (10 mM) were made in distilled water. AlexaFluor488-anti-human P-selectin antibody (Cat# 304916, RRID:AB_10719839) was purchased from Biolegend (San Diego, CA, USA). AlexaFluor647-annexin V was purchased from Thermo Fisher Scientific (Waltham, MA, USA). Firefly luciferin-luciferase mixture (CHRONO-LUME^®^), type I collagen from equine tendon suspension (CHRONO-PAR^®^ reagent), and ATP standard were purchased from Chrono-log Corp. (Haverton, PA, USA). PPACK (H-D-Phe-Pro-Arg-chloromethylketone, 2HCl), acetylsalicylic acid (ASA) and BSA were purchased from Merck (Darmstadt, Germany).

### 4.9. Statistical Analysis

All data were analyzed using GraphPad Prism 9.3.0 (GraphPad Software, San Diego, CA, USA). Blood pressure results are presented as means ± SEM from *n* experiments. The individual rat was considered the experimental unit (*n*) in each study. Normality was assessed with the Kolmogorov–Smirnov test. A *t*-test for unpaired data was used to compare baseline values between WKY and SHR groups. A *t*-test for paired data was used to compare the mean values when comparing to the baseline values, i.e., before vs. after CBG or its vehicle administration. To compare the effects of the vehicle and different doses of CBG as well as effects of hypertension on changes in BP and HR, a one-way analysis of variance (ANOVA) was performed followed by Bonferroni’s post hoc test.

Results from vessels studies are expressed as percent relaxation of the precontraction induced by phenylephrine. Sigmoidal CRCs were fitted using:

Y = Bottom + (Top − Bottom)/(1 + 10^(LogEC^_50_^−X)^)

where X is the logarithm of agonist concentration (M) and Y is the percent response. Potency (pEC_50_ = −log EC_50_) and maximal response (R_max_, at 300 μM CBG; the highest concentration of CBG) were derived from the curves [31]. For normally distributed data, one-way ANOVA followed by Dunnett’s post hoc test or Student’s t-test were applied as appropriate. The post hoc tests were performed only when the F-test was significant. Differences were considered significant at *p* < 0.05.

## 5. Conclusions

In summary, our study provides new insights into the effect of CBG on BP regulation in both normotensive and hypertensive conditions. Administration of CBG (at a dose of 10 mg/kg) effectively lowered BP in SHRs and their normotensive (WKY rats) controls and the magnitude of these responses (except for the decrease in SBP) was similar in both strains. Furthermore, studies on pithed and vagotomised rats support the notion that the blood pressure-lowering effect of CBG may involve both central and peripheral mechanisms (the vasorelaxant effect of CBG on the sMAs and the aorta is particularly significant); however, further research is needed to determine the exact sites of action of CBG in the cardiovascular system. Moreover, our preliminary in vitro study demonstrates, for the first time, that CBG, alone and in combination with CBD, reduced early platelet responses, as well as thrombus formation under flow.

Taken together, these data support further investigation of CBG as a cardiovascularly active compound. Our study combines an acute in vivo model in normotension and hypertension, an attempt to distinguish between peripheral and central mechanisms, and studies of platelet function in human whole blood under arterial flow conditions. Such a combination has not been described for CBG to date. However, chronic treatment studies, bleeding-risk assessment, receptor-level mechanistic experiments, and studies in hypertensive human subjects or using blood samples from hypertensive patients would be needed to determine whether CBG may represent a therapeutic strategy for conditions characterized by elevated blood pressure, vasoconstriction, or thromboembolic disorders.

## 6. Limitations and Perspectives

Our research expands our understanding of the effects of CBG on BP and vascular function in a model of hypertension, as well as on platelet function in vitro in human blood, which may be important for further research on CBG as a cardiovascularly active compound. However, before considering it as a therapeutic option for conditions associated with elevated blood pressure, vasoconstriction, or hypercoagulability, the transient nature of the response, the lack of studies on chronic use, the potential for tolerance development, and differences compared to more clinically plausible routes of administration than *i.v.* should be considered.

A limitation of our study is the lack of pharmacokinetic data. In our experiment, CBG was administered *i.v.*, and we observed hypotensive effects immediately after each dose. This route of administration is advantageous for assessing acute effects, as it ensures full systemic availability; however, it does not replace the measurement of plasma concentrations. CBG undergoes distribution and elimination, and these processes may vary among strains. A complete pharmacokinetic profile following *i.v.* administration of CBG at doses of 1–10 mg/kg is derived from studies in mice, where CBG at the highest dose exhibits a large volume of distribution and terminal phase half-life of approximately 10 h [32]. In rats, *i.v.* data are scarce: following administration of CBG (1.5 mg/animal), a large volume of distribution, relatively high clearance, and a half-life of approximately 2.5 h were observed [33]. Without pharmacokinetic data in our studies, it is unknown what concentrations were achieved after each dose of CBG or whether the concentration-time curve differed between WKY and SHR. Additionally, it cannot be determined whether the in vivo effects and differences between strains resulted solely from pharmacodynamics or also from differences in pharmacokinetics.

As CBG does not exhibit psychoactive properties and has been considered a legally accessible cannabinoid in certain jurisdictions, interest in its use for medical purposes has contributed to the development of a CBG market for CBG for medical purposes [5]; therefore, understanding the effects of long-term CBG use appears particularly important. The observed hypotensive, vasorelaxant, and antiplatelet effects of CBG may pose a potential clinical risk. An immediate drop in blood pressure with full availability of CBG (i.e., after *i.v.* administration) could theoretically suggest a risk of hypotension; however, it should be noted that the bioavailability of CBG after oral administration ranges from approximately 4% in an emulsion to 12% in oil [33]. Therefore, it appears that the risk of hypotension is unlikely with typical oral use. However, the risk may change with high doses, a high-fat meal, or formulations that enhance absorption [34]. Additionally, the antiplatelet effect raises concerns about a potential risk of bleeding when used concomitantly with antiplatelet drugs. Based on our data, we can only indicate that such a risk exists; however, a safety assessment requires studies on chronic exposure and a thorough evaluation of the mechanisms of action of CBG. It would be advisable to investigate the effects of long-term treatment. Repeated dosing could lead to metabolic induction or tissue accumulation, and result in a different cardiovascular profile than a single intravenous dose. Furthermore, although our findings complement existing knowledge regarding the putative mechanisms of action of CBG, it seems reasonable to investigate them further, which could contribute to a better assessment of CBG’s safety profile. It would be particularly important to determine whether the observed effects after CBG administration depend on receptor mechanisms, including cannabinoid receptor type 1, 2 (CB_1_, CB_2_), peroxisome proliferator-activated receptor gamma (PPARγ), transient receptor potential vanilloid subfamily member 1 (TRPV1), and α_2_-AR, for which CBG has documented pharmacological activity and which may account for these effects [5].

Additionally, the assessment of platelet function was carried out in vitro, which does not allow for a full evaluation of the CBG’s effect on the vascular endothelium, a key component of the haemostatic system. Furthermore, in studies of platelet aggregation, acetylsalicylic acid was used as the reference antiplatelet drug. In ex vivo studies on isolated vessels, the reference vasorelaxant compound was not included in the quantitative comparison of maximum vasodilatory efficacy, which we consider to be a methodological limitation.

Moreover, as sex is one of the well-documented factors associated with the regulation of cardiovascular function [35], a limitation of this study is that it involved only male animals. Further studies involving both sexes are needed to determine potential sex-specific differences in the effects of CBG on the cardiovascular system.

## Figures and Tables

**Table 1:** Systolic (SBP), diastolic (DBP) and mean (MBP) blood pressure, and heart rate (HR) before administration of cannabigerol (CBG) or its vehicle in pentobarbital-anesthetized and in pithed and vagotomised normotensive Wistar–Kyoto (WKY) rats and spontaneously hypertensive rats (SHR). Data are presented as mean ± SEM of 6–11 rats; * *p* < 0.05; ** *p*< 0.01; *** *p*< 0.001 compared to the respective values in WKY rats (*t*-test for unpaired data).

Group		*n*	WKY				*n*	SHR			
			SBP [mmHg]	DBP [mmHg]	MBP [mmHg]	HR [bts./min]		SBP [mmHg]	DBP [mmHg]	MBP [mmHg]	HR [bts./min]
Anesthetized	before vehicle	11	111 ± 5	83 ± 4	97 ± 4	280 ± 9	11	152 ± 6 ***	108 ± 6 **	130 ± 6 ***	299 ± 11
	before CBG 1 mg/kg	6	111 ± 5	86 ± 3	99 ± 4	305 ± 13	6	155 ± 7 ***	121 ± 5 ***	138 ± 5 ***	311 ± 14
	before CBG 3 mg/kg	9	107 ± 3	77 ± 4	92 ± 3	291 ± 8	7	159 ± 9 ***	118 ± 7 ***	139 ± 7 ***	289 ± 15
	before CBG 10 mg/kg	6	112 ± 3	87 ± 4	100 ± 3	277 ± 17	8	159 ± 9 ***	113 ± 7 **	135 ± 8 **	289 ± 13
Pithed	before vehicle	6	60 ± 7	30 ± 2	44 ± 5	269 ± 6	8	69 ± 6	45 ± 4 **	57 ± 5	303 ± 19 *
	before CBG 10 mg/kg	9	67 ± 4	34 ± 1	46 ± 2	254 ± 5	9	75 ± 4	48 ± 3 **	60 ± 3 **	299 ± 13 **

## Data Availability

The original contributions presented in this study are included in the article. Further inquiries can be directed to the corresponding author.

## Abbreviations

The following abbreviations are used in this manuscript:

2-AG	2-arachidonoylglycerol
α_1_-AR	alpha 1 adrenoceptor
α_2_-AR	alpha 2 adrenoceptor
Ach	acetylcholine
AEA	anandamide
ATP	adenosine triphosphate
BP	blood pressure
CBD	cannabidiol
CBG	cannabigerol
CRC	cumulative concentration–response curve
DBP	diastolic blood pressure
ECS	endocannabinoid system
HR	heart rate
hPAs	human pulmonary arteries
i.p.	intraperitoneal injection
i.v.	intravenous injection
MBP	mean blood pressure
Phe	phenylephrine
PPP	platelet-poor plasma
PRP	platelet-rich plasma
SBP	systolic blood pressure
SHR	spontaneously hypertensive rats
sMAs	small mesenteric arteries
WKY	Wistar Kyoto rats

## References

[1] Remiszewski P., Malinowska B. (2022). Why Multitarget Vasodilatory (Endo)cannabinoids Are Not Effective as Antihypertensive Compounds after Chronic Administration: Comparison of Their Effects on Systemic and Pulmonary Hypertension. Pharmaceuticals.

[2] Jastrząb A., Jarocka-Karpowicz I., Skrzydlewska E. (2022). The Origin and Biomedical Relevance of Cannabigerol. Int. J. Mol. Sci..

[3] Cascio M.G., Gauson L.A., Stevenson L.A., Ross R.A., Pertwee R.G. (2010). Evidence that the plant cannabinoid cannabigerol is a highly potent alpha2-adrenoceptor agonist and moderately potent 5HT1A receptor antagonist. Br. J. Pharmacol..

[4] Vernail V.L., Bingaman S.S., Silberman Y., Raup-Konsavage W.M., Vrana K.E., Arnold A.C. (2022). Acute Cannabigerol Administration Lowers Blood Pressure in Mice. Front. Physiol..

[5] Krzyżewska A., Kloza M., Kozłowska H. (2025). Comprehensive mini-review: Therapeutic potential of cannabigerol—Focus on the cardiovascular system. Front. Pharmacol..

[6] Krzyżewska A., Kloza M., Kozłowski M., Galicka A., Kozłowska H. (2025). Ex vivo study of the vasorelaxant activity induced by cannabigerol in human pulmonary artery- the role of endothelium, sex and selected clinical factors. Biochem. Pharmacol..

[7] Formukong E.A., Evans A.T., Evans F.J. (1989). The inhibitory effects of cannabinoids, the active constituents of *Cannabis sativa* L. on human and rabbit platelet aggregation. J. Pharm. Pharmacol..

[8] Polanska H.H., Petrlakova K., Papouskova B., Hendrych M., Samadian A., Storch J., Babula P., Masarik M., Vacek J. (2023). Safety assessment and redox status in rats after chronic exposure to cannabidiol and cannabigerol. Toxicology.

[9] Kicman A., Toczek M. (2020). The Effects of Cannabidiol, a Non-Intoxicating Compound of Cannabis, on the Cardiovascular System in Health and Disease. Int. J. Mol. Sci..

[10] Weresa J., Pędzińska-Betiuk A., Mińczuk K., Malinowska B., Schlicker E. (2022). Why Do Marijuana and Synthetic Cannabimimetics Induce Acute Myocardial Infarction in Healthy Young People?. Cells.

[11] Wang A., Dey S., Subhan S., Patel J., Frishman W.H., Aronow W.S. (2025). Cardiovascular Effects of Cannabinoids. Cardiol. Rev..

[12] Dielis A.W., Smid M., Spronk H.M., Hamulyak K., Kroon A.A., ten Cate H., de Leeuw P.W. (2005). The prothrombotic paradox of hypertension: Role of the renin-angiotensin and kallikrein-kinin systems. Hypertension.

[13] Kakar P., Lip G.Y. (2007). Hypertension: Endothelial dysfunction, the prothrombotic state and antithrombotic therapy. Expert Rev. Cardiovasc. Ther..

[14] Toczek M., Schlicker E., Remiszewski P., Malinowska B. (2024). Function of Presynaptic Inhibitory Cannabinoid CB1 Receptors in Spontaneously Hypertensive Rats and Its Modification by Enhanced Endocannabinoid Tone. Int. J. Mol. Sci..

[15] Lerman L.O., Kurtz T.W., Touyz R.M., Ellison D.H., Chade A.R., Crowley S.D., Mattson D.L., Mullins J.J., Osborn J., Eirin A. (2019). Animal Models of Hypertension: A Scientific Statement From the American Heart Association. Hypertension.

[16] Jama H.A., Muralitharan R.R., Xu C., O’Donnell J.A., Bertagnolli M., Broughton B.R.S., Head G.A., Marques F.Z. (2022). Rodent models of hypertension. Br. J. Pharmacol..

[17] Kurz C., Baranowska U., Lupiński S., Göthert M., Malinowska B., Schlicker E. (2009). Urethane, but not pentobarbitone, attenuates presynaptic receptor function in rats: A contribution to the choice of anaesthetic. Br. J. Pharmacol..

[18] Proudman R.G.W., Akinaga J., Baker J.G. (2022). The signaling and selectivity of α-adrenoceptor agonists for the human α2A, α2B and α2C-adrenoceptors and comparison with human α1 and β-adrenoceptors. Pharmacol. Res. Perspect..

[19] Vavřínová A., Behuliak M., Vodička M., Bencze M., Ergang P., Vaněčková I., Zicha J. (2024). More efficient adaptation of cardiovascular response to repeated restraint in spontaneously hypertensive rats: The role of autonomic nervous system. Hypertens. Res..

[20] Bridges L.E., Williams C.L., Pointer M.A., Awumey E.M. (2011). Mesenteric artery contraction and relaxation studies using automated wire myography. J. Vis. Exp..

[21] Bernatova I. (2014). Endothelial dysfunction in experimental models of arterial hypertension: Cause or consequence?. Biomed. Res. Int..

[22] Stanley C.P., Hind W.H., Tufarelli C., O’Sullivan S.E. (2015). Cannabidiol causes endothelium-dependent vasorelaxation of human mesenteric arteries via CB1 activation. Cardiovasc. Res..

[23] Baranowska-Kuczko M., Kozłowska H., Kloza M., Sadowska O., Kozłowski M., Kusaczuk M., Kasacka I., Malinowska B. (2020). Vasodilatory effects of cannabidiol in human pulmonary and rat small mesenteric arteries: Modification by hypertension and the potential pharmacological opportunities. J. Hypertens..

[24] Morales P., Reggio P.H., Jagerovic N. (2017). An Overview on Medicinal Chemistry of Synthetic and Natural Derivatives of Cannabidiol. Front. Pharmacol..

[25] De Angelis V., Koekman A.C., Weeterings C., Roest M., de Groot P.G., Herczenik E., Maas C. (2014). Endocannabinoids control platelet activation and limit aggregate formation under flow. PLoS ONE.

[26] Kossakowski R., Schlicker E., Toczek M., Weresa J., Malinowska B. (2019). Cannabidiol Affects the Bezold-Jarisch Reflex via TRPV1 and 5-HT3 Receptors and Has Peripheral Sympathomimetic Effects in Spontaneously Hypertensive and Normotensive Rats. Front. Pharmacol..

[27] Mińczuk K., Schlicker E., Krzyżewska A., Malinowska B. (2025). Angiotensin 1-7 injected into the rat paraventricular nucleus of hypothalamus increases blood pressure and heart rate via various receptors. Neuropharmacology.

[28] Baranowska-Kuczko M., Kozłowska H., Kloza M., Kusaczuk M., Harasim-Symbor E., Biernacki M., Kasacka I., Malinowska B. (2021). Vasoprotective Endothelial Effects of Chronic Cannabidiol Treatment and Its Influence on the Endocannabinoid System in Rats with Primary and Secondary Hypertension. Pharmaceuticals.

[29] de Witt S.M., Swieringa F., Cavill R., Lamers M.M., van Kruchten R., Mastenbroek T., Baaten C., Coort S., Pugh N., Schulz A. (2014). Identification of platelet function defects by multi-parameter assessment of thrombus formation. Nat. Commun..

[30] Rasband W.S. (2016). ImageJ.

[31] Kenakin T.P. (2019). A Pharmacology Primer: Techniques for More Effective and Strategic Drug Discovery.

[32] Zagzoog A., Halter K., Ha N., Jones A.M., Andres R., Kim A., Michel D., Alcorn J., Laprairie R.B. (2025). Pharmacokinetics and pharmacodynamics of cannabigerol (CBG) in the C57BL/6Crl mouse. Front. Pharmacol..

[33] Ryšánek P., Jelínek P., Housar H., Kozlík P., Křížek T., Nováková A., Sklenárová M., Paulusová V., Merdita S., Arora M. (2025). Effect of Quantitative Structural Properties and Drug Formulation in Four Cannabinoids (Cannabidiol, Cannabigerol, Cannabichromene, and Cannabinol) on Their Lymphatic Transport after Enteral Administration in Rats. Mol. Pharm..

[34] Story G., Lee J., Cohen G., Rani A., Doherty J., Sela D.A. (2024). Impact of Dietary Fat and Oral Delivery System on Cannabigerol Pharmacokinetics in Adults. Cannabis Cannabinoid Res..

[35] Shufelt C.L., Pacheco C., Tweet M.S., Miller V.M. (2018). Sex-Specific Physiology and Cardiovascular Disease. Adv. Exp. Med. Biol..

